# 4-Chloro-*N*-(2,3-dimethyl­phen­yl)-2-methyl­benzene­sulfonamide

**DOI:** 10.1107/S1600536811037536

**Published:** 2011-09-17

**Authors:** Vinola Z. Rodrigues, Sabine Foro, B. Thimme Gowda, K. Shakuntala

**Affiliations:** a Department of Chemistry, Mangalore University, Mangalagangotri 574 199, Mangalore, India; b Institute of Materials Science, Darmstadt University of Technology, Petersenstrasse 23, D-64287 Darmstadt, Germany

## Abstract

The asymmetric unit of the title compound, C_15_H_16_ClNO_2_S, contains two independent moleules. The conformation of the N—H bonds are *anti* to the *ortho*-methyl groups in the sulfonyl benzene rings of both the mol­ecules, while the N—H bonds are *anti* to the *ortho*- and *meta*-methyl groups in the aniline ring of one of the mol­ecules and *syn* in the other. Furthermore, the torsion angles of the C—SO_2_—NH—C segments in the two mol­ecules of are −66.8 (3) and 70.3 (3)°. The sulfonyl and the aniline benzene rings are oriented at angles of 44.1 (1) and 39.7 (1)° in the two mol­ecules. In the crystal, pairs of N—H⋯O hydrogen bonds link the mol­ecules into dimers.

## Related literature

For the preparation of the title compound, see: Savitha & Gowda (2006 ▶). For hydrogen-bonding modes of sulfonamides, see; Adsmond & Grant (2001 ▶). For studies on the effects of substituents on the structures and other aspects of *N*-(ar­yl)-amides, see: Arjunan *et al.* (2004 ▶); Gowda *et al.* (2006 ▶), on *N*-(ar­yl)-methane­sulfonamides, see: Gowda *et al.* (2007 ▶) and on *N*-(ar­yl)-aryl­sulfonamides, see: Gelbrich *et al.* (2007 ▶); Perlovich *et al.* (2006 ▶); Gowda *et al.* (2010 ▶).
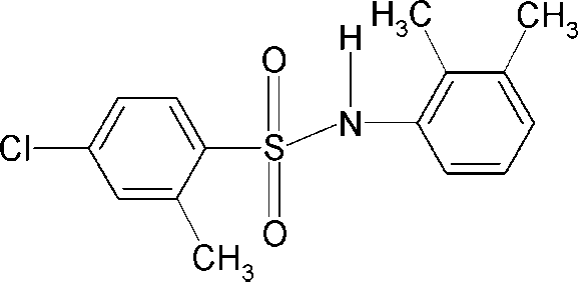


## Experimental

### 

#### Crystal data

C_15_H_16_ClNO_2_S
*M* *_r_* = 309.80
Triclinic, 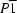

*a* = 8.2747 (7) Å
*b* = 11.0464 (9) Å
*c* = 17.021 (1) Å
α = 82.722 (7)°
β = 79.529 (7)°
γ = 80.267 (7)°
*V* = 1500.5 (2) Å^3^
*Z* = 4
Mo *K*α radiation
μ = 0.39 mm^−1^
*T* = 293 K
0.40 × 0.28 × 0.14 mm

#### Data collection

Oxford Diffraction Xcalibur diffractometer with a Sapphire CCD detector
Absorption correction: multi-scan (*CrysAlis RED*; Oxford Diffraction, 2009 ▶) *T*_min_ = 0.858, *T*_max_ = 0.947
10437 measured reflections
6064 independent reflections
4140 reflections with *I* > 2σ(*I*)
*R*_int_ = 0.016

#### Refinement

*R*[*F* ^2^ > 2σ(*F* ^2^)] = 0.066
*wR*(*F* ^2^) = 0.166
*S* = 1.12
6064 reflections
373 parameters
2 restraints
H atoms treated by a mixture of independent and constrained refinement
Δρ_max_ = 0.58 e Å^−3^
Δρ_min_ = −0.36 e Å^−3^

### 

Data collection: *CrysAlis CCD* (Oxford Diffraction, 2009 ▶); cell refinement: *CrysAlis RED* (Oxford Diffraction, 2009 ▶); data reduction: *CrysAlis RED*; program(s) used to solve structure: *SHELXS97* (Sheldrick, 2008 ▶); program(s) used to refine structure: *SHELXL97* (Sheldrick, 2008 ▶); molecular graphics: *PLATON* (Spek, 2009 ▶); software used to prepare material for publication: *SHELXL97*.

## Supplementary Material

Crystal structure: contains datablock(s) I, global. DOI: 10.1107/S1600536811037536/bq2304sup1.cif

Structure factors: contains datablock(s) I. DOI: 10.1107/S1600536811037536/bq2304Isup2.hkl

Supplementary material file. DOI: 10.1107/S1600536811037536/bq2304Isup3.cml

Additional supplementary materials:  crystallographic information; 3D view; checkCIF report

## Figures and Tables

**Table 1:** Hydrogen-bond geometry (Å, °)

*D*—H⋯*A*	*D*—H	H⋯*A*	*D*⋯*A*	*D*—H⋯*A*
N1—H1*N*⋯O3	0.85 (2)	2.15 (2)	2.971 (4)	162 (4)
N2—H2*N*⋯O2	0.85 (2)	2.12 (2)	2.954 (4)	166 (4)

**Fig. 1.**
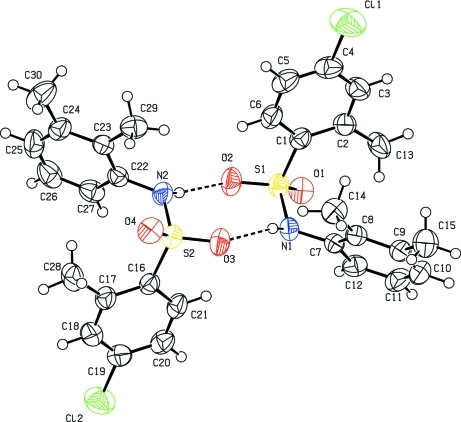
Molecular structure of the title compound, showing the atom labelling scheme and displacement ellipsoids are drawn at the 50% probability level.

**Fig. 2.**
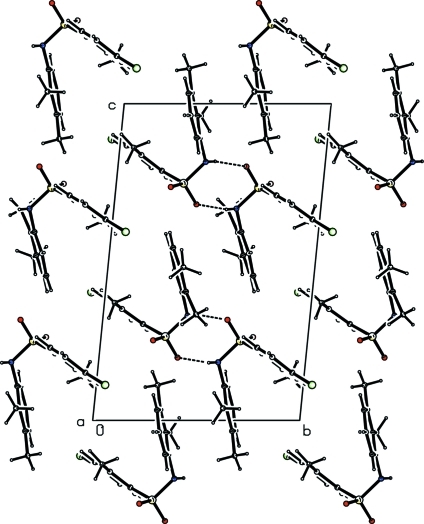
Molecular packing of (I) with hydrogen bonding shown as dashed lines.

## supplementary crystallographic information

### Comment

The amide and sulfonamide moieties are the constituents of many biologically significant compounds. The hydrogen bonding preferences of sulfonamides have been investigated (Adsmond & Grant, 2001). As part of our work on the substituent effects on the structures and other aspects of *N*-(aryl)-amides (Arjunan *et al.*, 2004; Gowda *et al.*, 2006), *N*-(aryl)-methanesulfonamides (Gowda *et al.*, 2007) and *N*-(aryl)-arylsulfonamides (Gowda *et al.*, 2010), in the present work, the crystal structure of 4-Chloro-2-methyl-*N*- (2,3-dimethylphenyl)benzenesulfonamide (I) has been determined (Fig. 1).

The asymmetric unit of (I) contains two independent moleules. The conformation of the N—H bonds are *anti* to the *ortho*-methyl groups in the sulfonyl benzene rings of both the molecules, while, the N—H bonds are *anti* to the *ortho*- and *meta*-methyl groups in the anilino benzene ring of one of the molecules and *syn* in the other.

The torsion angles of the C—SO_2_—NH—C segments in the two molecules of (I) are -66.8 (3)° and 70.3 (3)°, compared to the values of -61.9 (4)° and 69.7 (4)° in the two independent molecules of 4-chloro-2-methyl- *N*-(phenyl)-benzenesulfonamide (II) and -76.5 (5)° and -48.3 (4)° in 4-chloro-2-methyl-*N*-(4-methylphenyl)-benzenesulfonamide (III) (Gowda *et al.*, 2010).

The sulfonyl and the aniline benzene rings in (I) are tilted relative to each other by 44.1 (1)° in molecule 1 and 39.7 (1)° in molecule 2, compared to the values of 86.6 (2)° and 83.0 (2)° in the two independent molecules of (II), and 76.6 (2)° in molecule 1 and 70.7 (2)° in molecule 2 of (III).

The other bond parameters in (I) are similar to those observed in (II), (III) and other aryl sulfonamides (Perlovich *et al.*, 2006; Gelbrich *et al.*, 2007).

In the crystal, the intermolecular N–H···O hydrogen bonds (Table 1) link the molecules as dimers (Fig. 2).

### Experimental

The solution of *m*-chlorotoluene (10 ml) in chloroform (40 ml) was treated dropwise with chlorosulfonic acid (25 ml) at 0 ° C. After the initial evolution of hydrogen chloride subsided, the reaction mixture was brought to room temperature and poured into crushed ice in a beaker. The chloroform layer was separated, washed with cold water and allowed to evaporate slowly. The residual 2-methyl-4-chlorobenzenesulfonylchloride was treated with 2,3-dimethylaniline in the stoichiometric ratio and boiled for ten minutes. The reaction mixture was then cooled to room temperature and added to ice cold water (100 cc). The resultant solid 4-chloro-2-methyl-*N*- (2,3-dimethylphenyl)-benzenesulfonamide was filtered under suction and washed thoroughly with cold water. It was then recrystallized to constant melting point from dilute ethanol. The purity of the compound was checked and characterized by recording its infrared and NMR spectra (Savitha & Gowda, 2006).

Prism like light pink single crystals used in X-ray diffraction studies were grown in ethanolic solution by slow evaporation at room temperature.

### Refinement

The H atoms of the NH groups were located in a difference map and later restrained to N—H = 0.86 (2) %A. The other H atoms were positioned with idealized geometry using a riding model with the aromatic C—H = 0.93Å and methyl C—H = 0.96 Å. All H atoms were refined with isotropic displacement parameters. The *U*_iso_(H) values were set at 1.2*U*_eq_(C-aromatic, N) and 1.5*U*_eq_(*C*-methyl).

### Crystal data

**Table:**

C_15_H_16_ClNO_2_S	*Z* = 4
*M**_r_* = 309.80	*F*(000) = 648
Triclinic, *P*1	*D*_x_ = 1.371 Mg m^−^^3^
Hall symbol: -P 1	Mo *K*α radiation, λ = 0.71073 Å
*a* = 8.2747 (7) Å	Cell parameters from 3482 reflections
*b* = 11.0464 (9) Å	θ = 2.5–27.9°
*c* = 17.021 (1) Å	µ = 0.39 mm^−^^1^
α = 82.722 (7)°	*T* = 293 K
β = 79.529 (7)°	Prism, light pink
γ = 80.267 (7)°	0.40 × 0.28 × 0.14 mm
*V* = 1500.5 (2) Å^3^	

### Data collection

**Table:**

Oxford Diffraction Xcalibur diffractometer with a Sapphire CCD detector	6064 independent reflections
Radiation source: fine-focus sealed tube	4140 reflections with *I* > 2σ(*I*)
graphite	*R*_int_ = 0.016
Rotation method data acquisition using ω scans	θ_max_ = 26.4°, θ_min_ = 2.5°
Absorption correction: multi-scan (*CrysAlis RED*; Oxford Diffraction, 2009)	*h* = −8→10
*T*_min_ = 0.858, *T*_max_ = 0.947	*k* = −13→13
10437 measured reflections	*l* = −20→21

### Refinement

**Table:**

Refinement on *F*^2^	Primary atom site location: structure-invariant direct methods
Least-squares matrix: full	Secondary atom site location: difference Fourier map
*R*[*F*^2^ > 2σ(*F*^2^)] = 0.066	Hydrogen site location: inferred from neighbouring sites
*wR*(*F*^2^) = 0.166	H atoms treated by a mixture of independent and constrained refinement
*S* = 1.12	*w* = 1/[σ^2^(*F*_o_^2^) + (0.0505*P*)^2^ + 1.8811*P*] where *P* = (*F*_o_^2^ + 2*F*_c_^2^)/3
6064 reflections	(Δ/σ)_max_ = 0.001
373 parameters	Δρ_max_ = 0.58 e Å^−^^3^
2 restraints	Δρ_min_ = −0.36 e Å^−^^3^

### Special details

**Table:**

Geometry. All e.s.d.'s (except the e.s.d. in the dihedral angle between two l.s. planes) are estimated using the full covariance matrix. The cell e.s.d.'s are taken into account individually in the estimation of e.s.d.'s in distances, angles and torsion angles; correlations between e.s.d.'s in cell parameters are only used when they are defined by crystal symmetry. An approximate (isotropic) treatment of cell e.s.d.'s is used for estimating e.s.d.'s involving l.s. planes.
Refinement. Refinement of *F*^2^ against ALL reflections. The weighted *R*-factor *wR* and goodness of fit *S* are based on *F*^2^, conventional *R*-factors *R* are based on *F*, with *F* set to zero for negative *F*^2^. The threshold expression of *F*^2^ > σ(*F*^2^) is used only for calculating *R*-factors(gt) *etc*. and is not relevant to the choice of reflections for refinement. *R*-factors based on *F*^2^ are statistically about twice as large as those based on *F*, and *R*- factors based on ALL data will be even larger.

### Fractional atomic coordinates and isotropic or equivalent isotropic displacement parameters (Å^2^)

**Table:**

	*x*	*y*	*z*	*U*_iso_*/*U*_eq_	
Cl1	0.52306 (16)	1.04270 (13)	0.11188 (11)	0.1031 (5)	
S1	0.01951 (12)	0.67472 (8)	0.25114 (5)	0.0460 (2)	
O1	−0.1489 (3)	0.7348 (2)	0.26297 (15)	0.0552 (7)	
O2	0.0856 (4)	0.6027 (2)	0.31763 (14)	0.0610 (7)	
N1	0.0393 (4)	0.5780 (3)	0.18362 (17)	0.0443 (7)	
H1N	0.134 (3)	0.533 (3)	0.182 (2)	0.053*	
C1	0.1528 (4)	0.7859 (3)	0.2117 (2)	0.0437 (8)	
C2	0.1054 (4)	0.8924 (3)	0.1619 (2)	0.0494 (9)	
C3	0.2245 (5)	0.9684 (3)	0.1318 (3)	0.0590 (10)	
H3	0.1971	1.0392	0.0982	0.071*	
C4	0.3823 (5)	0.9412 (4)	0.1508 (3)	0.0597 (10)	
C5	0.4282 (5)	0.8370 (4)	0.2000 (3)	0.0608 (11)	
H5	0.5349	0.8195	0.2127	0.073*	
C6	0.3138 (5)	0.7598 (4)	0.2299 (2)	0.0519 (9)	
H6	0.3436	0.6888	0.2629	0.062*	
C7	−0.0170 (4)	0.6186 (3)	0.10819 (19)	0.0388 (7)	
C8	0.0950 (4)	0.6401 (3)	0.0383 (2)	0.0422 (8)	
C9	0.0315 (5)	0.6788 (3)	−0.0338 (2)	0.0495 (9)	
C10	−0.1381 (5)	0.6941 (4)	−0.0326 (3)	0.0591 (10)	
H10	−0.1795	0.7191	−0.0803	0.071*	
C11	−0.2468 (5)	0.6733 (4)	0.0370 (3)	0.0597 (11)	
H11	−0.3607	0.6858	0.0366	0.072*	
C12	−0.1861 (4)	0.6338 (3)	0.1073 (2)	0.0501 (9)	
H12	−0.2589	0.6172	0.1545	0.060*	
C13	−0.0679 (5)	0.9320 (4)	0.1388 (3)	0.0660 (12)	
H13A	−0.1452	0.9589	0.1848	0.079*	
H13B	−0.1029	0.8634	0.1206	0.079*	
H13C	−0.0641	0.9986	0.0967	0.079*	
C14	0.2798 (4)	0.6235 (4)	0.0383 (2)	0.0569 (10)	
H14A	0.3052	0.5743	0.0864	0.068*	
H14B	0.3141	0.7028	0.0362	0.068*	
H14C	0.3376	0.5830	−0.0076	0.068*	
C15	0.1476 (6)	0.7027 (4)	−0.1115 (2)	0.0740 (13)	
H15A	0.2166	0.6269	−0.1253	0.089*	
H15B	0.2163	0.7615	−0.1052	0.089*	
H15C	0.0839	0.7348	−0.1534	0.089*	
Cl2	−0.09225 (15)	−0.06815 (12)	0.40474 (9)	0.0840 (4)	
S2	0.39623 (11)	0.31016 (8)	0.26485 (5)	0.0461 (2)	
O3	0.3263 (3)	0.3796 (2)	0.19847 (15)	0.0607 (7)	
O4	0.5651 (3)	0.2535 (2)	0.25283 (15)	0.0579 (7)	
N2	0.3740 (4)	0.4077 (3)	0.33141 (18)	0.0475 (7)	
H2N	0.282 (3)	0.456 (3)	0.334 (2)	0.057*	
C16	0.2681 (4)	0.1958 (3)	0.3046 (2)	0.0424 (8)	
C17	0.3215 (5)	0.0878 (3)	0.3519 (2)	0.0489 (9)	
C18	0.2057 (5)	0.0090 (3)	0.3816 (2)	0.0558 (10)	
H18	0.2368	−0.0631	0.4133	0.067*	
C19	0.0459 (5)	0.0349 (3)	0.3652 (2)	0.0518 (9)	
C20	−0.0065 (5)	0.1399 (4)	0.3189 (2)	0.0556 (10)	
H20	−0.1145	0.1562	0.3077	0.067*	
C21	0.1047 (4)	0.2201 (4)	0.2893 (2)	0.0511 (9)	
H21	0.0706	0.2924	0.2585	0.061*	
C22	0.4406 (4)	0.3718 (3)	0.4052 (2)	0.0404 (7)	
C23	0.6040 (4)	0.3884 (3)	0.4066 (2)	0.0408 (8)	
C24	0.6675 (5)	0.3519 (3)	0.4783 (2)	0.0510 (9)	
C25	0.5644 (6)	0.3059 (4)	0.5452 (2)	0.0649 (12)	
H25	0.6061	0.2827	0.5929	0.078*	
C26	0.4023 (6)	0.2936 (4)	0.5431 (2)	0.0649 (12)	
H26	0.3358	0.2638	0.5891	0.078*	
C27	0.3396 (5)	0.3256 (3)	0.4727 (2)	0.0524 (9)	
H27	0.2309	0.3164	0.4703	0.063*	
C28	0.4975 (5)	0.0499 (4)	0.3714 (3)	0.0690 (12)	
H28A	0.5307	0.1174	0.3918	0.083*	
H28B	0.5729	0.0287	0.3235	0.083*	
H28C	0.4994	−0.0201	0.4111	0.083*	
C29	0.7049 (5)	0.4485 (4)	0.3345 (2)	0.0571 (10)	
H29A	0.6317	0.5008	0.3023	0.069*	
H29B	0.7720	0.3861	0.3033	0.069*	
H29C	0.7754	0.4971	0.3517	0.069*	
C30	0.8441 (5)	0.3647 (5)	0.4836 (3)	0.0803 (15)	
H30A	0.8636	0.4473	0.4642	0.096*	
H30B	0.9199	0.3073	0.4515	0.096*	
H30C	0.8610	0.3476	0.5385	0.096*	

### Atomic displacement parameters (Å^2^)

**Table:**

	*U*^11^	*U*^22^	*U*^33^	*U*^12^	*U*^13^	*U*^23^
Cl1	0.0637 (8)	0.0905 (9)	0.1610 (15)	−0.0338 (7)	−0.0213 (8)	−0.0007 (9)
S1	0.0517 (6)	0.0501 (5)	0.0345 (4)	−0.0050 (4)	−0.0070 (4)	−0.0013 (4)
O1	0.0483 (15)	0.0622 (16)	0.0507 (15)	−0.0037 (12)	0.0005 (12)	−0.0073 (12)
O2	0.0770 (19)	0.0663 (17)	0.0369 (13)	−0.0031 (14)	−0.0143 (13)	0.0025 (12)
N1	0.0492 (18)	0.0428 (17)	0.0386 (15)	−0.0028 (13)	−0.0090 (14)	0.0018 (13)
C1	0.0432 (19)	0.0454 (19)	0.0437 (19)	−0.0010 (15)	−0.0113 (16)	−0.0107 (15)
C2	0.044 (2)	0.0425 (19)	0.064 (2)	−0.0018 (16)	−0.0161 (18)	−0.0065 (17)
C3	0.053 (2)	0.044 (2)	0.082 (3)	−0.0069 (18)	−0.020 (2)	−0.001 (2)
C4	0.044 (2)	0.060 (2)	0.079 (3)	−0.0130 (18)	−0.011 (2)	−0.016 (2)
C5	0.044 (2)	0.070 (3)	0.074 (3)	0.000 (2)	−0.022 (2)	−0.023 (2)
C6	0.050 (2)	0.057 (2)	0.052 (2)	−0.0012 (18)	−0.0198 (18)	−0.0066 (18)
C7	0.0441 (19)	0.0356 (17)	0.0376 (17)	−0.0076 (14)	−0.0076 (15)	−0.0030 (14)
C8	0.0436 (19)	0.0409 (18)	0.0429 (19)	−0.0085 (15)	−0.0064 (15)	−0.0050 (15)
C9	0.067 (3)	0.046 (2)	0.0381 (19)	−0.0141 (18)	−0.0113 (17)	−0.0037 (15)
C10	0.070 (3)	0.057 (2)	0.057 (2)	−0.005 (2)	−0.031 (2)	−0.0062 (19)
C11	0.049 (2)	0.064 (3)	0.073 (3)	−0.0065 (19)	−0.025 (2)	−0.014 (2)
C12	0.045 (2)	0.054 (2)	0.053 (2)	−0.0141 (17)	−0.0053 (17)	−0.0083 (17)
C13	0.050 (2)	0.045 (2)	0.102 (3)	−0.0053 (18)	−0.030 (2)	0.017 (2)
C14	0.046 (2)	0.071 (3)	0.051 (2)	−0.0146 (19)	0.0008 (17)	−0.0053 (19)
C15	0.097 (4)	0.081 (3)	0.044 (2)	−0.026 (3)	−0.006 (2)	0.003 (2)
Cl2	0.0711 (8)	0.0751 (8)	0.1086 (10)	−0.0281 (6)	−0.0147 (7)	0.0035 (7)
S2	0.0475 (5)	0.0498 (5)	0.0412 (5)	−0.0046 (4)	−0.0127 (4)	−0.0012 (4)
O3	0.0714 (18)	0.0662 (17)	0.0443 (14)	−0.0072 (14)	−0.0201 (13)	0.0061 (13)
O4	0.0504 (16)	0.0676 (17)	0.0542 (16)	−0.0069 (13)	−0.0039 (12)	−0.0093 (13)
N2	0.0470 (18)	0.0454 (17)	0.0506 (17)	0.0005 (13)	−0.0177 (15)	−0.0029 (14)
C16	0.046 (2)	0.0422 (19)	0.0406 (18)	−0.0005 (15)	−0.0127 (15)	−0.0086 (15)
C17	0.049 (2)	0.045 (2)	0.054 (2)	0.0033 (16)	−0.0197 (18)	−0.0062 (17)
C18	0.060 (2)	0.044 (2)	0.064 (2)	−0.0040 (18)	−0.017 (2)	−0.0008 (18)
C19	0.051 (2)	0.051 (2)	0.056 (2)	−0.0090 (17)	−0.0104 (18)	−0.0110 (18)
C20	0.043 (2)	0.065 (3)	0.063 (2)	−0.0058 (18)	−0.0175 (19)	−0.007 (2)
C21	0.046 (2)	0.056 (2)	0.051 (2)	0.0028 (17)	−0.0205 (17)	−0.0029 (17)
C22	0.0439 (19)	0.0348 (17)	0.0418 (18)	−0.0046 (14)	−0.0056 (15)	−0.0050 (14)
C23	0.0441 (19)	0.0369 (17)	0.0418 (18)	−0.0088 (14)	−0.0051 (15)	−0.0044 (14)
C24	0.056 (2)	0.048 (2)	0.052 (2)	0.0047 (17)	−0.0185 (18)	−0.0169 (17)
C25	0.098 (4)	0.052 (2)	0.041 (2)	0.010 (2)	−0.022 (2)	−0.0056 (18)
C26	0.091 (3)	0.053 (2)	0.043 (2)	−0.012 (2)	0.008 (2)	0.0007 (18)
C27	0.055 (2)	0.049 (2)	0.052 (2)	−0.0155 (17)	0.0028 (18)	−0.0066 (17)
C28	0.052 (2)	0.055 (2)	0.098 (3)	0.0012 (19)	−0.032 (2)	0.015 (2)
C29	0.050 (2)	0.063 (2)	0.060 (2)	−0.0185 (19)	−0.0006 (19)	−0.0093 (19)
C30	0.061 (3)	0.098 (4)	0.090 (3)	0.011 (3)	−0.035 (3)	−0.036 (3)

### Geometric parameters (Å, °)

**Table:**

Cl1—C4	1.739 (4)	Cl2—C19	1.738 (4)
S1—O1	1.429 (3)	S2—O4	1.421 (3)
S1—O2	1.436 (2)	S2—O3	1.438 (2)
S1—N1	1.635 (3)	S2—N2	1.626 (3)
S1—C1	1.777 (4)	S2—C16	1.776 (4)
N1—C7	1.438 (4)	N2—C22	1.445 (4)
N1—H1N	0.850 (18)	N2—H2N	0.848 (18)
C1—C6	1.396 (5)	C16—C21	1.398 (5)
C1—C2	1.403 (5)	C16—C17	1.405 (5)
C2—C3	1.387 (5)	C17—C18	1.388 (5)
C2—C13	1.533 (5)	C17—C28	1.530 (5)
C3—C4	1.377 (5)	C18—C19	1.376 (5)
C3—H3	0.9300	C18—H18	0.9300
C4—C5	1.376 (6)	C19—C20	1.371 (5)
C5—C6	1.368 (5)	C20—C21	1.371 (5)
C5—H5	0.9300	C20—H20	0.9300
C6—H6	0.9300	C21—H21	0.9300
C7—C12	1.384 (5)	C22—C27	1.388 (5)
C7—C8	1.390 (5)	C22—C23	1.400 (5)
C8—C9	1.410 (5)	C23—C24	1.403 (5)
C8—C14	1.509 (5)	C23—C29	1.500 (5)
C9—C10	1.382 (5)	C24—C25	1.389 (6)
C9—C15	1.509 (5)	C24—C30	1.512 (6)
C10—C11	1.372 (6)	C25—C26	1.378 (6)
C10—H10	0.9300	C25—H25	0.9300
C11—C12	1.374 (5)	C26—C27	1.374 (6)
C11—H11	0.9300	C26—H26	0.9300
C12—H12	0.9300	C27—H27	0.9300
C13—H13A	0.9600	C28—H28A	0.9600
C13—H13B	0.9600	C28—H28B	0.9600
C13—H13C	0.9600	C28—H28C	0.9600
C14—H14A	0.9600	C29—H29A	0.9600
C14—H14B	0.9600	C29—H29B	0.9600
C14—H14C	0.9600	C29—H29C	0.9600
C15—H15A	0.9600	C30—H30A	0.9600
C15—H15B	0.9600	C30—H30B	0.9600
C15—H15C	0.9600	C30—H30C	0.9600
			
O1—S1—O2	119.33 (16)	O4—S2—O3	119.65 (17)
O1—S1—N1	108.10 (16)	O4—S2—N2	107.93 (16)
O2—S1—N1	104.79 (15)	O3—S2—N2	104.94 (16)
O1—S1—C1	109.28 (16)	O4—S2—C16	109.10 (16)
O2—S1—C1	107.43 (17)	O3—S2—C16	106.85 (16)
N1—S1—C1	107.27 (16)	N2—S2—C16	107.82 (16)
C7—N1—S1	120.3 (2)	C22—N2—S2	120.5 (2)
C7—N1—H1N	117 (3)	C22—N2—H2N	118 (3)
S1—N1—H1N	109 (3)	S2—N2—H2N	112 (3)
C6—C1—C2	120.4 (3)	C21—C16—C17	120.1 (3)
C6—C1—S1	116.4 (3)	C21—C16—S2	116.5 (3)
C2—C1—S1	123.2 (3)	C17—C16—S2	123.3 (3)
C3—C2—C1	117.3 (3)	C18—C17—C16	117.0 (3)
C3—C2—C13	117.5 (3)	C18—C17—C28	118.0 (3)
C1—C2—C13	125.2 (3)	C16—C17—C28	124.9 (3)
C4—C3—C2	121.4 (4)	C19—C18—C17	121.6 (4)
C4—C3—H3	119.3	C19—C18—H18	119.2
C2—C3—H3	119.3	C17—C18—H18	119.2
C5—C4—C3	121.2 (4)	C20—C19—C18	121.6 (4)
C5—C4—Cl1	120.4 (3)	C20—C19—Cl2	119.5 (3)
C3—C4—Cl1	118.4 (3)	C18—C19—Cl2	119.0 (3)
C6—C5—C4	118.7 (4)	C19—C20—C21	118.1 (3)
C6—C5—H5	120.7	C19—C20—H20	120.9
C4—C5—H5	120.7	C21—C20—H20	120.9
C5—C6—C1	121.1 (4)	C20—C21—C16	121.5 (3)
C5—C6—H6	119.5	C20—C21—H21	119.2
C1—C6—H6	119.5	C16—C21—H21	119.2
C12—C7—C8	121.2 (3)	C27—C22—C23	122.2 (3)
C12—C7—N1	117.8 (3)	C27—C22—N2	119.2 (3)
C8—C7—N1	121.1 (3)	C23—C22—N2	118.6 (3)
C7—C8—C9	118.2 (3)	C22—C23—C24	117.9 (3)
C7—C8—C14	121.7 (3)	C22—C23—C29	120.8 (3)
C9—C8—C14	120.1 (3)	C24—C23—C29	121.3 (3)
C10—C9—C8	119.3 (4)	C25—C24—C23	118.9 (4)
C10—C9—C15	120.3 (4)	C25—C24—C30	120.3 (4)
C8—C9—C15	120.4 (4)	C23—C24—C30	120.8 (4)
C11—C10—C9	121.7 (4)	C26—C25—C24	122.2 (4)
C11—C10—H10	119.2	C26—C25—H25	118.9
C9—C10—H10	119.2	C24—C25—H25	118.9
C10—C11—C12	119.4 (4)	C27—C26—C25	119.6 (4)
C10—C11—H11	120.3	C27—C26—H26	120.2
C12—C11—H11	120.3	C25—C26—H26	120.2
C11—C12—C7	120.2 (4)	C26—C27—C22	119.0 (4)
C11—C12—H12	119.9	C26—C27—H27	120.5
C7—C12—H12	119.9	C22—C27—H27	120.5
C2—C13—H13A	109.5	C17—C28—H28A	109.5
C2—C13—H13B	109.5	C17—C28—H28B	109.5
H13A—C13—H13B	109.5	H28A—C28—H28B	109.5
C2—C13—H13C	109.5	C17—C28—H28C	109.5
H13A—C13—H13C	109.5	H28A—C28—H28C	109.5
H13B—C13—H13C	109.5	H28B—C28—H28C	109.5
C8—C14—H14A	109.5	C23—C29—H29A	109.5
C8—C14—H14B	109.5	C23—C29—H29B	109.5
H14A—C14—H14B	109.5	H29A—C29—H29B	109.5
C8—C14—H14C	109.5	C23—C29—H29C	109.5
H14A—C14—H14C	109.5	H29A—C29—H29C	109.5
H14B—C14—H14C	109.5	H29B—C29—H29C	109.5
C9—C15—H15A	109.5	C24—C30—H30A	109.5
C9—C15—H15B	109.5	C24—C30—H30B	109.5
H15A—C15—H15B	109.5	H30A—C30—H30B	109.5
C9—C15—H15C	109.5	C24—C30—H30C	109.5
H15A—C15—H15C	109.5	H30A—C30—H30C	109.5
H15B—C15—H15C	109.5	H30B—C30—H30C	109.5
			
O1—S1—N1—C7	50.9 (3)	O4—S2—N2—C22	−47.4 (3)
O2—S1—N1—C7	179.2 (3)	O3—S2—N2—C22	−176.0 (3)
C1—S1—N1—C7	−66.8 (3)	C16—S2—N2—C22	70.3 (3)
O1—S1—C1—C6	151.7 (3)	O4—S2—C16—C21	−154.0 (3)
O2—S1—C1—C6	20.8 (3)	O3—S2—C16—C21	−23.3 (3)
N1—S1—C1—C6	−91.4 (3)	N2—S2—C16—C21	89.0 (3)
O1—S1—C1—C2	−31.0 (3)	O4—S2—C16—C17	28.5 (3)
O2—S1—C1—C2	−161.8 (3)	O3—S2—C16—C17	159.2 (3)
N1—S1—C1—C2	86.0 (3)	N2—S2—C16—C17	−88.4 (3)
C6—C1—C2—C3	0.5 (5)	C21—C16—C17—C18	0.0 (5)
S1—C1—C2—C3	−176.7 (3)	S2—C16—C17—C18	177.4 (3)
C6—C1—C2—C13	−178.9 (4)	C21—C16—C17—C28	178.8 (4)
S1—C1—C2—C13	3.8 (5)	S2—C16—C17—C28	−3.8 (5)
C1—C2—C3—C4	−0.7 (6)	C16—C17—C18—C19	0.4 (6)
C13—C2—C3—C4	178.7 (4)	C28—C17—C18—C19	−178.5 (4)
C2—C3—C4—C5	0.3 (7)	C17—C18—C19—C20	−0.1 (6)
C2—C3—C4—Cl1	−178.9 (3)	C17—C18—C19—Cl2	−179.9 (3)
C3—C4—C5—C6	0.3 (6)	C18—C19—C20—C21	−0.6 (6)
Cl1—C4—C5—C6	179.6 (3)	Cl2—C19—C20—C21	179.2 (3)
C4—C5—C6—C1	−0.5 (6)	C19—C20—C21—C16	1.0 (6)
C2—C1—C6—C5	0.1 (6)	C17—C16—C21—C20	−0.7 (6)
S1—C1—C6—C5	177.5 (3)	S2—C16—C21—C20	−178.3 (3)
S1—N1—C7—C12	−76.7 (4)	S2—N2—C22—C27	−92.8 (4)
S1—N1—C7—C8	104.2 (3)	S2—N2—C22—C23	89.1 (4)
C12—C7—C8—C9	0.5 (5)	C27—C22—C23—C24	2.7 (5)
N1—C7—C8—C9	179.5 (3)	N2—C22—C23—C24	−179.3 (3)
C12—C7—C8—C14	−179.8 (3)	C27—C22—C23—C29	−174.8 (3)
N1—C7—C8—C14	−0.8 (5)	N2—C22—C23—C29	3.2 (5)
C7—C8—C9—C10	0.0 (5)	C22—C23—C24—C25	−2.6 (5)
C14—C8—C9—C10	−179.7 (3)	C29—C23—C24—C25	174.8 (3)
C7—C8—C9—C15	−179.6 (3)	C22—C23—C24—C30	178.7 (3)
C14—C8—C9—C15	0.7 (5)	C29—C23—C24—C30	−3.8 (5)
C8—C9—C10—C11	0.4 (6)	C23—C24—C25—C26	0.8 (6)
C15—C9—C10—C11	−179.9 (4)	C30—C24—C25—C26	179.5 (4)
C9—C10—C11—C12	−1.4 (6)	C24—C25—C26—C27	1.1 (6)
C10—C11—C12—C7	1.8 (6)	C25—C26—C27—C22	−1.1 (6)
C8—C7—C12—C11	−1.4 (5)	C23—C22—C27—C26	−0.8 (5)
N1—C7—C12—C11	179.5 (3)	N2—C22—C27—C26	−178.9 (3)

### Hydrogen-bond geometry (Å, °)

**Table:**

*D*—H···*A*	*D*—H	H···*A*	*D*···*A*	*D*—H···*A*
N1—H1N···O3	0.85 (2)	2.15 (2)	2.971 (4)	162 (4)
N2—H2N···O2	0.85 (2)	2.12 (2)	2.954 (4)	166 (4)
